# Hábitos de vida saludables, sistema musculoesquelético y su impacto en la población trabajadora en la pospandemia

**DOI:** 10.15446/rsap.V25n3.104127

**Published:** 2023-05-01

**Authors:** Wilder Alfonso Hernández-Duarte

**Affiliations:** 1 WH: FT. M. Sc. Salud y Seguridad en el Trabajo. Corporación Universitaria Minuto de Dios, Uniminuto. Bogotá, Colombia. whernandezd@uniminuto.edu.co Corporación Universitaria Minuto de Dios Corporación Universitaria Minuto de Dios Bogotá Colombia whernandezd@uniminuto.edu.co

**Keywords:** COVID-19, dolor musculoesquelético, trastornos de traumas acumulados, conducta sedentaria, factor de riesgo, promoción de la salud, prevención de enfermedades *(fuente: DeCS, BIREME)*, COVID-19, musculoskeletal pain, cumulative trauma disorders, sedentary behavior, risk factors, health promotion, disease prevention *(source: MeSH, NLM)*

## Abstract

Se presenta una reflexión sobre posibles efectos de la pandemia para la población trabajadora en cuanto a los desórdenes musculoesqueléticos, el incremento de la conducta sedentaria por el confinamiento y sus consecuencias, dado el inicio de actividades laborales de manera habitual por parte de las organizaciones a raíz del comportamiento de la pandemia por la COVID-19. Por tanto, sería relevante generar las respectivas medidas en torno a las condiciones de trabajo, analizar su repercusión sobre las condiciones de salud y las consecuencias que puede representar para la empresa y el sector sanitario. Es importante fomentar su debida gestión de manera predictiva y en el contexto de la prevención de la enfermedad y la promoción de la salud en los lugares de trabajo por parte de las organizaciones, con respaldo en políticas públicas.

## Abstract (en)

A reflection is presented on possible effects of the pandemic for the working population in terms of musculoskeletal disorders, increased sedentary behavior due to confinement and its consequences, given the start of work activities on a regular basis by organizations as a result of the behavior of the pandemic by COVID-19. Therefore, it would be relevant to generate the respective measures on working conditions and analyze their impact on health conditions and the consequences they may represent for the company and the health sector. It is vital to promote its proper management in a predictive manner and in the context of disease prevention and health promotion in workplaces by organizations supported under public policies.

Al hacer mención de los hábitos de vida saludable, se entienden estos como aquellas pautas o comportamientos cotidianos de una persona apropiados como patrones de conducta que demuestran cierta consistencia en el tiempo y pueden constituir una condición de riesgo o de seguridad, dependiendo de su naturaleza o contexto. Estas conductas están enmarcadas en aquellas asociadas con consumo de alcohol, tabaco o psicoactivos, patrones nutricionales o sedentarismo, entre otros 1.

Los esquemas no adecuados de hábitos de vida saludable han demostrado su relación con enfermedades crónicas no transmisibles (ECNT). En el año 2021, la Organización Mundial de la Salud (OMS) 2 reportó a nivel mundial un total de 41 millones de muertes, de las cuales 15 millones se presentaron en el grupo etario de 30 a 69 años en países de ingresos bajos y en población en edad productiva. Además, las enfermedades cardiovasculares, seguidas del cáncer, las enfermedades respiratorias y la diabetes ocuparon los primeros lugares entre las causas de muerte, siendo el 80 % de estas prematuras 2.

A lo anterior se suma la contingencia sanitaria por la covid-19. Según estudios realizados, grupos poblacionales con antecedentes de estas ECNT presentan más susceptibilidad a mostrar cuadros graves por contagio e incluso muerte 3.

En el contexto del trabajo, el estrés de origen laboral se puede manifestar en diferentes niveles: fisiológico y conductual, entre otros 4. Por tanto, factores organizacionales inapropiados pueden fomentar hábitos nocivos para la salud del trabajador que, al combinarse con otros factores intralaborales, pueden agravar los efectos del estrés y por supuesto deteriorar su salud 5.

## Desórdenes musculoesqueléticos y hábitos de vida saludable

En cuanto a los desórdenes musculoesqueléticos y su relación con los hábitos de vida saludable en el contexto laboral, la Organización Internacional del Trabajo (OIT)^5^ plantea que, en el trabajador, al exponerse a estos factores organizacionales generadores de estrés laboral, el sistema musculoesquelético también se puede ver afectado.

Así mismo, se ha descrito que personas con índice de masa corporal (IMC) elevado experimentan problemas a nivel de la región lumbar. La población con esta característica presenta inestabilidad en la zona mencionada, toda vez que soporta un peso excesivo, como también dificultad para la movilidad y propensión a una fatiga muscular más temprana 6,7. Además, se ha planteado que en personas con niveles bajos de actividad física los tejidos conectivos no cuentan con condiciones óptimas para soportar exigencias que pueden generar las tareas laborales 8, por lo que se ha evidenciado que personas que realizan actividad física con regularidad generan un factor protector para evitar el dolor lumbar 9. Adicionalmente, se encuentra en estudio si en las personas que consumen tabaco, al generarse vasoconstricción en los vasos sanguíneos, se reduce el flujo sanguíneo, por lo cual la oxigenación a los tejidos musculares y a los demás tejidos conectivos se reduce, y además se altera la calidad del colágeno 9.

En el contexto laboral, trabajadores de diversas actividades o sectores económicos pueden presentar situaciones en las cuales el sedentarismo, por ejemplo, puede ser uno de los factores de riesgo que apoyen la generación de algún desorden musculoesqueléticos (DME), a parte de los factores intralaborales.

Vicente *et al.*^10^ sostienen que a medida que la población trabajadora envejece, es más posible que aumenten los casos de dolor lumbar, debido a la influencia de factores individuales, laborales y hábitos de vida. Se recomienda, por tanto, que se incentiven los estudios al respecto. Los investigadores en mención 10 indagaron sobre la influencia de factores sociales y ocupacionales en trabajadores con dolor lumbar y detectaron que en personas con trabajos operativos o manuales la intensidad del dolor era alta, pero el impacto de la incapacidad era más fuerte entre trabajadores de oficina, principalmente mujeres, probablemente por la baja exigencia física asociada con el trabajo de oficina y similares.

En actividades económicas con un nivel de riesgo particular, como la seguridad pública, la población de uniformados también ha reportado esta situación. En un análisis sobre el ausentismo en los periodos de enero a octubre del 2019, en una unidad en la ciudad de Cali (Colombia), y también sobre las pruebas físicas aplicadas a la población trabajadora, se encontró que la mayoría de los diagnósticos médicos por los cuales se generaba la ausencia tenía relación con el sistema musculoesquelético. El tronco y los miembros inferiores fueron las zonas corporales más afectadas y un 24 % de la población con antecedentes de ausentismo presentaba algún grado de sobrepeso u obesidad. Se identificaron relaciones estadísticas significativas entre la zona afectada y el IMC, así como con la edad 11.

Esta situación también se ha detectado entre profesionales del área de la salud. Se llevó a cabo un análisis de factores de riesgo asociados con DME en miembros superiores en una entidad sanitaria de régimen especial ubicada en Bogotá (Colombia), para identificar su relación con las áreas de medicina, fisioterapia, enfermería, odontología y bacteriología. Además de algunos factores intralaborales con los cuales se detectaron relaciones significativas y las áreas descritas, se identificaron relaciones significativas con una escasa ejecución de actividad física entre los profesionales de todas las áreas 12.

## Su impacto en postpandemia

Si bien se han reportado estudios en los cuales hábitos como el sedentarismo han tenido alguna participación previa a la pandemia, es necesario tener presente los efectos de este componente una vez se inicie el proceso de retorno a las actividades habituales. Arocha 13, en su revisión de la literatura menciona que gracias a los avances tecnológicos en el contexto laboral, así como a los nuevos modos de trabajo aplicados como el teletrabajo, no se requiere el mismo esfuerzo físico que en otros tiempos, lo que facilita que surjan complicaciones en diferentes sistemas como el musculoesquelético. Este aspecto pudo incrementarse a raíz de la pandemia y las medidas sanitaras aplicadas para evitar la propagación desmedida de la infección generada por el SARS-CoV-2.

Chatkoff *et al*.^14^ consideran que, a raíz de la pandemia, se hicieron cambios en diferentes contextos como el fomento del trabajo en casa, lo cual ha afectado a pacientes con dolor crónico, entre otros, al reducirse el nivel de actividad física que se hacía regularmente, con lo cual se pueden incrementar situaciones de ansiedad. En su estudio, se detectó una relación inversa entre la actividad física y el estado de ánimo negativo en población con antecedentes de dolor crónico.

Si se revisa el contexto laboral, se han reportado antecedentes sobre los efectos de la pandemia en diferentes ámbitos para el trabajador, y el dolor musculoesquelético no es la excepción. El trabajo en casa puede propiciar este tipo de dolencias a partir de las características irregulares de los puestos de trabajo organizados para tal propósito, así como aspectos organizacionales dados por los tiempos prolongados 15 y la reducción de la actividad física 13.

En España, Carpintero *et al.*^16^ hicieron un análisis de sintomatología musculoesquelética y factores asociados, a nivel nacional, por medio de una comparación antes y durante el periodo de confinamiento de la pandemia. Así, identificaron relaciones estadísticamente significativas entre dolor musculoesquelético e IMC antes de la pandemia, y la intensidad del dolor se incrementó durante el periodo de confinamiento. Se identificó un incremento en los índices de sedentarismo durante el confinamiento, lo que repercute en las condiciones de trofismo del sistema musculoesquelético, como se mencionó anteriormente, y aumento de la sintomatología osteomuscular.

Según lo relacionado en este escrito, hábitos poco saludables como el sedentarismo han mostrado una relación no solo con las ECNT, sino con la generación de problemas osteomusculares antes de iniciar la pandemia. Con el confinamiento, este comportamiento ha podido mostrar un incremento entre la población en general y puede propiciar una serie de complicaciones en lo relacionado con el trabajo, las condiciones de trabajo y sus efectos sobre las condiciones de salud. Dado que la COVID-19 viene presentando un comportamiento hacia el descenso, y que muchos países vienen desarrollando sus procesos de retorno a la normalidad, sería recomendable para las organizaciones iniciar programas orientados a la detección temprana de estos efectos entre la población y aplicar medidas anticipatorias en el marco de la gestión de la seguridad y la salud en el trabajo. Esto implicaría, entre otros aspectos, la revisión o actualización de los exámenes médicos ocupacionales para conocer el estado en el cual retornarían sus colaboradores, la actualización de las condiciones de trabajo a partir de los ajustes o cambios hechos por la empresa para mantenerse activos durante la contingencia sanitaria y la determinación de acciones para considerarse en el marco de la prevención de la enfermedad y la gestión de la promoción de la salud en los entornos laborales, con el ánimo de evitar efectos sobre las organizaciones y mayor carga sobre el sistema de salud ♠
